# Valproate induces non-hyperammonemic encephalopathy below a standard serum concentration range: A case report

**DOI:** 10.1097/MD.0000000000043228

**Published:** 2025-07-11

**Authors:** Tomohiro Uemura, Nana Suzuki, Jun Souma, Hikaru Kamiya, Shiori Takeguchi-Kikuchi, Tsukasa Saito, Jun Sawada, Naoki Nakagawa

**Affiliations:** a Division of Respiratory Medicine and Neurology, Department of Internal Medicine, Asahikawa Medical University, Asahikawa, Hokkaido, Japan; b Division of Cardiology and Nephrology, Department of Internal Medicine, Asahikawa Medical University, Asahikawa, Hokkaido, Japan.

**Keywords:** carnitine, consciousness disturbance, encephalopathy, non-hyperammonemic, valproate, valproate concentration

## Abstract

**Rationale:**

Valproate (VPA)-induced encephalopathy may present with consciousness disturbance, epilepsy, and psychiatric disorders after acute or long-term VPA use. VPA-induced encephalopathy typically occurs in the setting of either or both hyperammonemia and low serum L-carnitine levels, however, it may also develop without hyperammonemia or decreased serum L-carnitine levels.

**Patient concerns:**

We herein describe a 71-year-old male with VPA-induced nonhyperammonemic encephalopathy with a history of long-term VPA therapy for a corticosteroid-induced mood disorder. He presented with persistent consciousness disturbance.

**Diagnoses:**

Serum levels of ammonia and carnitine were within the normal ranges, while that of VPA was below the standard range. A cerebral spinal fluid analysis revealed elevated protein with a normal white cell count. Mild brain atrophy was observed on brain magnetic resonance images and mild periventricular hyperintensity on fluid-attenuated inversion recovery images. Electroencephalography showed semirhythmic delta activity in the parieto-occipital area, and sharp transients in the bifrontal area. The patient was diagnosed with VPA-induced nonhyperammonemic encephalopathy.

**Interventions:**

The patient was discontinued from VPA.

**Outcomes:**

After the discontinuation of VPA, the patient’s consciousness was gradually restored, and electroencephalographic findings also improved. After discharge, he has not had consciousness disturbance, or obvious cognitive impairment.

**Lessons:**

VPA-induced encephalopathy needs to be considered when patients treated with VPA exhibit neurological symptoms, even if their serum concentration of VPA is below the standard range, and they do not have hyperammonemia or carnitine deficiency. In such cases, treatment with VPA needs to be ceased immediately because neurological symptoms may be alleviated by its discontinuation.

## 1. Introduction

Valproate (VPA) is an antiepileptic drug (AED) that is broadly used to treat generalized or focal forms of epilepsy. It is also used as a mood stabilizer in patients with psychiatric conditions, such as bipolar and schizoaffective disorders, and as a prophylaxis for migraine.^[1]^

VPA is tolerated well and is associated with only mild adverse reactions, including gastrointestinal conditions (nausea, vomiting, and diarrhea), nervous system symptoms (sedation, ataxia, and tremor), weight gain, and hair loss.^[2]^ VPA-induced encephalopathy is a rare and serious complication, occurring only in 0.1 to 2.5% of patients after the acute or long-term use of VPA.^[3]^ The manifestations of the acute type include consciousness disturbance, epilepsy, and psychiatric disorders,^[4,5]^ while the chronic type causes cognitive impairment, Parkinsonism, and autism.^[6–8]^ Furthermore, most patients develop either or both hyperammonemia and low serum L-carnitine levels. We herein present a case of VPA-induced non-hyperammonemic encephalopathy with a normal serum level of carnitine below the standard range of VPA. VPA-induced encephalopathy needs to be considered when patients treated with VPA exhibit neurological symptoms, even if their serum concentration of VPA is below the standard range, and they do not have hyperammonemia or carnitine deficiency. In such cases, treatment with VPA needs to be ceased immediately because neurological symptoms may be alleviated by its discontinuation.

## 2. Case report

A 71-year-old man was diagnosed with granulomatosis with polyangiitis 9 years prior to admission. The patient was orally administered prednisolone and methotrexate. He developed subglottic stenosis caused by the exacerbation of granulomatous polyangiitis 2 years later, and underwent tracheotomy. At the same time, he developed a headache, and a cerebrospinal fluid (CSF) analysis showed elevated total protein and a normal mononuclear cell count. Brain enhanced magnetic resonance imaging revealed hypertrophy and enhancement of the dura mater. Since pachymeningitis caused by granulomatosis with polyangiitis was considered, the prednisolone dosage was increased. Headache subsequently improved, and total protein in CSF decreased (from 96.4 to 76.8 mg/dL). However, he developed irritability and restlessness due to the corticosteroid-induced mood disorder. The administration of VPA and lithium carbonate attenuated psychological symptoms, and the dosage of prednisolone was reduced. The patient exhibited signs of bradykinesia and rigidity of the limbs 3 years prior to admission. Dopamine transporter imaging with single-photon emission computed tomography revealed low uptake in the striatum bilaterally. The administration of L-dopa alleviated these symptoms; therefore, the patient was diagnosed with Parkinson disease. He had no obvious cognitive impairment, fluctuation of consciousness, hallucination, or rapid eye movement sleep behavior disorder. Furthermore, the patient had no previous history of epilepsy or liver disease. The family history was unremarkable, and his parents were not consanguineous.

Approximately 40 days before admission, the patient was diagnosed with sciatica, developed pain in the right leg, and was initially treated with mirogabalin besylate and nonsteroidal anti-inflammatory drugs 1 month prior to admission. However, leg pain persisted; therefore, these medications were discontinued and replaced with tramadol hydrochloride 5 days before admission. One day later, the patient manifested consciousness disturbance, which persisted even after the withdrawal of tramadol hydrochloride, and thus, he was admitted to our hospital. The patient’s family had been managing his medication before he was admitted to our hospital. Therefore, it was unlikely that he inadvertently interrupted treatment with medications, including VPA, or skipped some doses.

Administered drugs and their daily oral dosages on admission are shown in Table 1. The patient’s height and weight were 167 cm and 64.1 kg, respectively. Vital signs upon hospitalization were as follows: blood pressure, 166/98 mm Hg; pulse rate, 90 beats per minute; and body temperature, 37.2°C. His oxygen saturation level was 98% in ambient air. Physical findings of the chest, abdomen, and extremities were within the normal ranges. A neurological examination showed somnolence and disorientation. The patient was unable to respond appropriately and had difficulty communicating. Bilateral light reflex responses were prompt, and left patellar and Achilles tendon reflex responses were increased. Laboratory examinations revealed the following results (Table 2). Liver and renal functions and electrolyte levels were normal. Ammonia was within the normal range. Serum concentrations of VPA and lithium were below the standard range. T-carnitine, F-carnitine, acylcarnitine, lactate, and pyruvate levels were all within normal ranges. The total protein concentration in CSF was high, while the cell count was within the normal range. Proinflammatory markers, such as the immunoglobulin G index, IL-6, IL-10, and oligoclonal bands in CSF, did not indicate any pathological conditions. CSF levels of lactate and pyruvate were within the standard ranges, while serum and CSF levels of glutamine were not measured. The Tau protein, phosphorylated tau protein, and amyloid beta 42/40 ratio in CSF were within the standard ranges.

**Table 1:** Administered drugs on admission.

	Daily oral dosages
Valproate	600 mg
Lithium carbonate	500 mg
Levodopa	200 mg
Aripiprazole	3 mg
Ramelteon	8 mg
Prednisolone	2 mg
Methotrexate	10 mg (per wk)
Folic acid	5 mg (per wk)
Rabeprazole	10 mg
Rosuvastatin	5 mg
Amphotericin B	400 mg
Sulfamethoxazole	400 mg
Trimethoprim	80 mg
Carbocysteine	1500 mg
Ambroxol	45 mg
D-chlorpheniramine maleate	6 mg
Edoxaban	30 mg
Shakuyakukanzoto	5 g
Alendronate sodium hydrate	35 mg (per wk)

**Table 2:** Laboratory findings.

		Reference value
Urinalysis		
Protein	Negative	
Occult blood	Negative	
Peripheral blood		
White blood cells	6810	(3300–8600/μL)
Red blood cells	544	(435-555 × 10^4^/μL)
Hemoglobin	17.9	(13.7–16.8 g/dL)
Platelet count	15.1	(15.8-34.8 × 10^4^/μL)
Blood chemistry		
Total protein	6.5	(6.6–8.1 g/dL)
Albumin	4.0	(4.1–5.1 g/dL)
Urea nitrogen	10.3	(8.0–20.0 mg/dL)
Creatinine	1.00	(0.65–1.07 mg/dL)
Sodium	141	(138–145 mmol/L)
Potassium	4.2	(3.6–4.8 mmol/L)
Cl	105	(101–108 mmol/L)
C-reactive protein	1.46	(<0.14 mg/dL)
Total bilirubin	1.4	(0.4–1.5 mg/dL)
AST	15	(13–30 U/L)
ALT	12	(10–42 U/L)
ALP	86	(38–113 U/L)
γ-GTP	16	(13–64 U/L)
ammonia	36	(20–70 mg/dL)
HDL cholesterol	59.4	(40.0–90.0 mg/dL)
LDL cholesterol	76.0	(65.0–139.0 mg/dL)
Triglycerides	59.0	(40–149 mg/dL)
glucose	131	(73–109 mg/dL)
Hemoglobin A1c (NGSP)	6.1	(4.9–6.0%)
VPA	19.33	(50–100 mg/dL)
Lithium	0.01	(0.3–1.0 mEq/L)
T-Carnitine	62.2	(45–91 mmol/L)
F-Carnitine	54.3	(36–74 mmol/L)
Acylcarnitine	7.9	(6–23 mmol/L)
Lactate	9.3	(3.7–16.3 mg/dL)
Pyruvate	0.76	(0.30–0.90 mg/dL)
Free triiodothyronine	3.09	(11.0–31.0 pg/mL)
Free thyroxine	1.27	(2.30–4.00 ng/dL)
Thyroid-stimulating hormone	1.31	(0.50–5.00 µIU/mL)
Immunology		
PR3 ANCA	1.5	(<2.0 IU/mL)
Amphiphysin Ab	Negative	
CV2 Ab	Negative	
Ma2/Ta Ab	Negative	
Ri Ab	Negative	
Yo Ab	Negative	
Hu Ab	Negative	
Recoverin Ab	Negative	
SOX1 Ab	Negative	
Titin Ab	Negative	
Zic4 Ab	Negative	
Glutamic acid decarboxylase Ab	Negative	
Tr (DNER) Ab	Negative	
Cerebrospinal fluid		
Appearance	Translucent	
Pressure	150	(30–180 mmH_2_O)
Cells	1	(0–3/μL)
Neutrophils	0	(%)
Lymphocytes	100	(%)
Protein	112.6	(10–40 mg/dL)
Immunoglobulin G	7.4	(0.5–4 mg/dL)
Immunoglobulin G index	0.51	<0.7
Oligo clonal bands	Negative	
Lactate	16.2	(13.7–20.5 mg/dL)
Pyruvate	1.02	(0.63–0.77 mg/dL)
Glucose	63	(mg/dL)
(Serum glucose)	(133)	(mg/dL)
IL-6	3.2	(<4.3 pg/mL)
IL-10	<2	(<4 pg/mL)
Tau protein	178	(<200 pg/mL)
Phosphorylated tau protein	<25.0	(21.5–59.0 pg/mL)
Amyloid beta 42/40 ratio	0.125	(>0.067)
Bacterial culture	Negative	
Mycobacterium culture	Negative	
HSV DNA	Negative	
VZV DNA	Negative	
CMV DNA	Negative	
EBV DNA	Negative	
HHV-6 DNA	Negative	
HHV-7 DNA	Negative	
Cytology	Negative	

Ab = antibody, ALT = alanine aminotransferase, AST = aspartate aminotransferase, CMV = cytomegalovirus, DNER = delta/notch-like epidermal growth factor-related receptor, EBV = Epstein-Barr virus, HDL = high-density lipoprotein, HHV = human herpesvirus, HSV = human simplex virus, IL-10 = interleukin-10, IL-6 = interleukin-6, LDL = low-density lipoprotein, NGSP = national glycohemoglobin standardization program, VPA = valproate, VZV = varicella-zoster virus, γ-GTP = γ-glutamyl transpeptidase.

**Figure 1.**
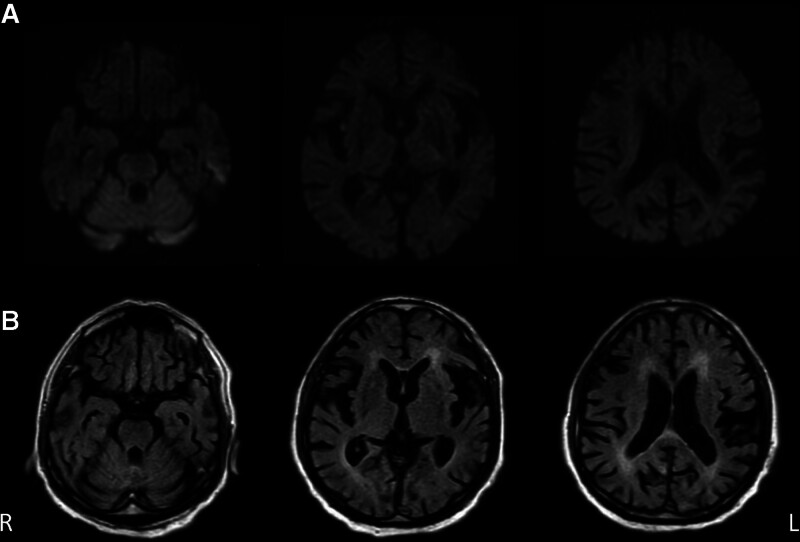
Brain magnetic resonance imaging (MRI) on admission. (A) Diffusion-weighted and (B) fluid-attenuated inversion recovery (FLAIR) images. Brain MRI showed mild atrophy of brain. FLAIR images indicated mild periventricular hyperintensity.

**Figure 2.**
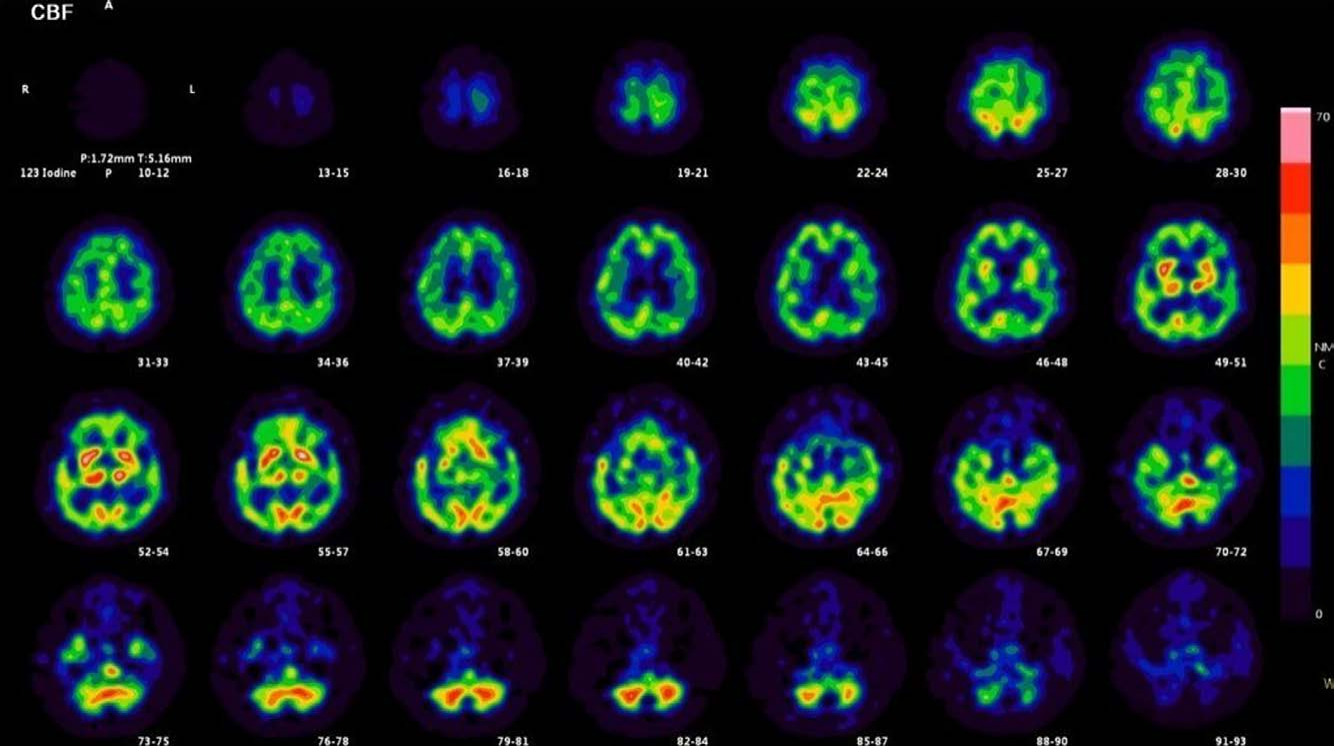
Brain perfusion single-photon emission computed tomography (^123^I-IMP). Low blood flow was observed in the posterior parietal lobe as well as in the parietal and, frontal cortices, suggesting diffuse function impairment of the brain due to encephalopathy.

**Figure 3.**
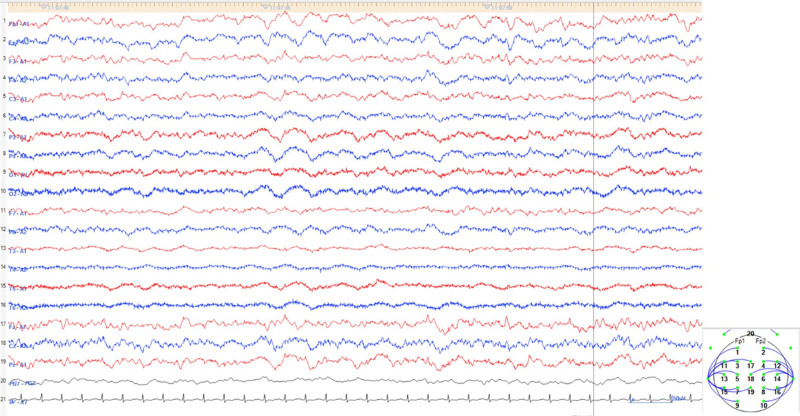
Electroencephalography (EEG). EEG showed semirhythmic delta activity in the parieto-occipital area, and sharp transients in the bifrontal area.

Brain magnetic resonance imaging revealed mild brain atrophy, and fluid-attenuated inversion recovery (FLAIR) images showed mild periventricular hyperintensity (Fig. 1). Brain perfusion single-photon emission computed tomography revealed low blood flow in the posterior parietal lobe and in the parietal and frontal cortices (Fig. 2). Electroencephalography (EEG) showed semirhythmic delta activity in the parieto-occipital area and sharp transients in the bifrontal area (Fig. 3). No tumors were detected by truncal computed tomography.

**Figure 4.**
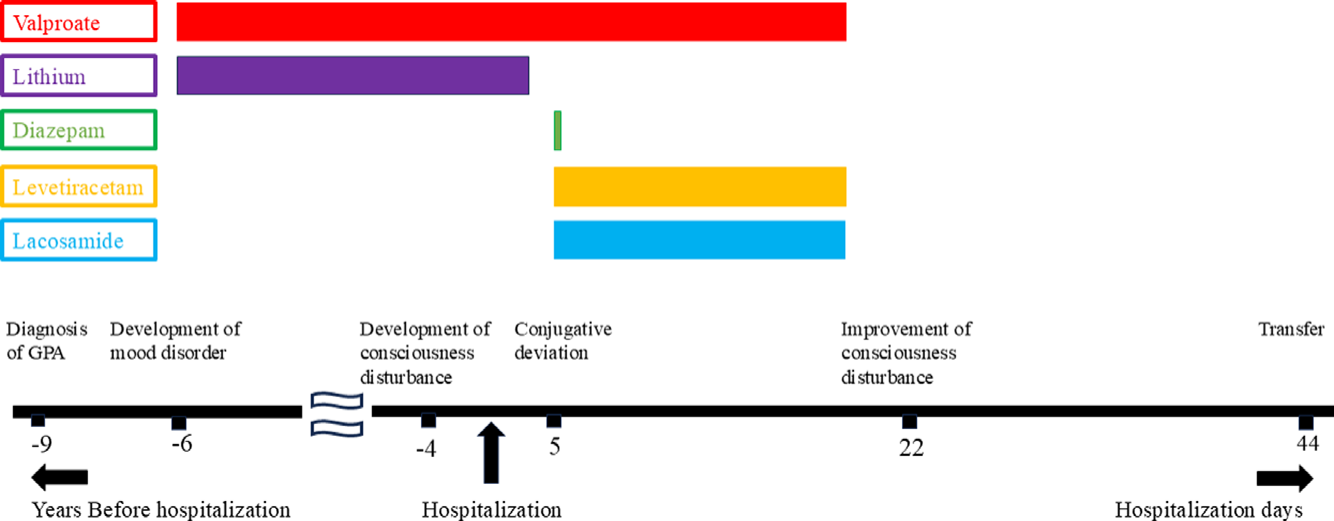
Timeline of the clinical course and administered drugs. GPA = granulomatous polyangiitis.

We suspected that consciousness disturbance was a side effect of lithium carbonate or aripiprazole and, thus, ceased administering these medications on the third day of hospitalization. However, the condition persisted. Administered drugs on admission remained at the same dosages, except for rosuvastatin, amphotericin B, trimethoprim, d-chlorpheniramine maleate, Shakuyakukanzoto, and alendronate sodium hydrate, which were discontinued. In addition, the patient presented with conjugative deviation on the fifth day. Since epilepsy was suspected, we administered diazepam (a single injection of 10 mg), followed by levetiracetam (500 mg/d) and lacosamide (started with 100 mg/d, then increased to 200 mg on the 14th day). However, the state of consciousness did not improve. We discontinued these AEDs and VPA to perform polysomnography under drug-free conditions on the 19th day. The patient gradually recovered consciousness around the 22nd day and was able to converse coherently and follow instructions around the 26th day. When we performed EEG after discontinuing all AED regimens, a background rhythm within a range of 8 to 9 Hz was observed with no general slow activity. Oral food intake resumed on the 30th day of hospitalization, and consciousness disturbance was no longer detected on the 38th day. The patient was transferred to another hospital for rehabilitation on the 44th day after admission and was discharged 3 months later (Fig. 4). Since then, he has not had consciousness disturbance, or obvious cognitive impairment (his score on the mini-mental state examination was 26/30 18 mo after discharge); therefore, he was unlikely to have Alzheimer disease or dementia with Lewy bodies. Moreover, autoimmune encephalopathy was ruled out because he recovered without increases in prednisolone or the addition of other immunotherapies. His symptoms were unlikely to be caused by the corticosteroid-induced mood disorder and catatonia because his mental condition remained stable before admission, and his symptoms did not improve with diazepam. No other causes of neurological symptoms were identified. Therefore, the patient was diagnosed with VPA-induced non-hyperammonemic encephalopathy.

## 3. Discussion

We herein present a rare case of VPA-induced encephalopathy without elevated serum levels of ammonia, carnitine, or VPA. VPA-induced morbidity is generally caused by hyperammonemia and L-carnitine deficiency. However, some individuals develop VPA-induced encephalopathy without hyperammonemia after the long-term oral administration of VPA.^[9]^

The pathogenesis of VPA-induced encephalopathy involves the enhanced absorption of glutamine, which facilitates the release of ammonia in renal glomeruli. Under conditions of increased serum ammonia levels, glutamate and ammonia are converted to glutamine by glutamine synthetase. Elevated glutamine levels in astrocytes may increase osmotic pressure, resulting in brain edema.^[3]^

Most patients with VPA-induced encephalopathy also have L-carnitine deficiency. VPA may promote the excretion of carnitine from renal glomeruli, followed by the formation of cellular compounds combined with carnitine and coenzyme A, subsequently inducing hypocarnitinemia. This impairs the metabolism of free fatty acids, resulting in increased blood ammonia levels and subsequent encephalopathy.^[3]^

Our patient presented with neither hyperammonemia nor L-carnitine deficiency; however, consciousness improved after the discontinuation of VPA. In addition, the blood concentration of VPA was below the standard range. Limited information is currently available on non-hyperammonemic VPA-induced encephalopathy with standard serum VPA levels.^[10–13]^ VPA-induced hyperammonemic encephalopathy within a standard range of blood VPA has also been reported.^[9,14,15]^

The mechanisms underlying non-hyperammonemic VPA-induced encephalopathy may include the following: increased glutamine production and suppressed glutamine release by astrocytes may accelerate the development of VPA-induced encephalopathy without hyperammonemia. Glutamine accumulation, followed by an increase in intracellular osmolarity, may induce astrocyte swelling and cerebral edema. In addition, the storage of VPA metabolites, 2-en-VPA and 4-en-VP, increases the risk of cerebral edema^[16,17]^; VPA directly affects the central nervous system and may increase the incidence of encephalopathy in the presence of normal ammonia levels^[18]^; As an AED, VPA may promote mitochondrial toxicity^[14]^ which may, in turn, induce non-hyperammonemia VPA-induced encephalopathy; VPA may activate the synthesis of free radicals, inducing oxidative stress and neuronal injury, increasing the risk of encephalopathy.^[19,20]^ Based on these findings, VPA may induce encephalopathy without increases in serum ammonia or VPA concentrations. Since serum and CSF levels of glutamine were not evaluated in our patient, we cannot speculate about the specific role of glutamine under this pathological condition. Further studies are required to evaluate the role of glutamine and elucidate the basis of its development and risk factors.

VPA-induced encephalopathy was described shortly after VPA was introduced in clinical practice, with the rapid resolution of encephalopathy following withdrawal of the drug.^[21]^ Previous studies showed that patients may develop VPA-induced encephalopathy after prolonged use of this drug.^[9]^ Galiza et al described a patient who developed VPA-induced encephalopathy 20 years after treatment with VPA.^[11]^ Our patient was diagnosed approximately 6 years after the onset of VPA therapy. Therefore, irrespective of the duration of VPA use, the possibility of VPA-induced encephalopathy needs to be considered during treatment.

Moreover, the combined use of VPA and specific drugs is associated with a higher risk of VPA-induced encephalopathy. Levetiracetam and topiramate have been reported to expedite the progression of this condition when administered in combination with VPA.^[3]^ Levy et al showed that lithium may contribute to the disease by elevating serum VPA levels and promoting hyperammonemia.^[22]^ In the present case, neurological symptoms were not alleviated for a long time after the discontinuation of lithium carbonate therapy. Therefore, lithium carbonate may not be involved in the pathogenesis of VPA-induced encephalopathy.

Consciousness disturbance is a common clinical manifestation in patients with VPA-induced encephalopathy that mainly manifests as drowsiness, lethargy, or coma.^[3]^ VPA-induced encephalopathy may present with unilateral or bilateral seizures and status epilepticus.^[3,17]^ EEG findings of VPA-induced encephalopathy are nonspecific, and it may present with diffuse slowing and a predominance of rhythmic theta and delta activity, triphasic waves, and occasional bursts of frontal intermittent rhythmic delta activities.^[17]^ These findings are reversible with the discontinuation of VPA.^[23]^ Since epilepsy was initially suspected in our patient, AEDs, including diazepam, levetiracetam, and lacosamide, were administered; however, his symptoms did not improve. After VPA was discontinued, his consciousness gradually recovered, and EEG findings improved. These findings suggest that his symptoms were caused by VPA-induced encephalopathy rather than by status epilepticus. In psychiatric patients with VPA-induced encephalopathy, reduced motor activity, lethargy, or confusion may be misattributed to the worsening of psychosis or a mood disorder, and also lead to an inappropriate increase in the VPA dose.^[24]^ Therefore, it is necessary to carefully consider whether the neurological symptoms of patients taking VPA are due to an exacerbation of the primary disease or VPA-induced encephalopathy.

Limited information is available on the white cell count and CSF protein levels in VPA-induced encephalopathy.^[11,25–27]^ CSF findings in VPA-induced encephalopathy were normal or unremarkable in these studies; however, details were not provided. The CSF analysis of the present case revealed elevated protein with normal cell counts. Although it is difficult to confirm this, the elevated CSF protein in our patient may have been caused by VPA-induced encephalopathy. Another possible reason is the effect of pachymeningitis due to granulomatosis with polyangiitis, although the disease remained stable.

Neurological symptoms caused by VPA-induced encephalopathy may be alleviated by the discontinuation of VPA. Nevertheless, the turnaround time and improvement rate differ among affected individuals.^[3]^ Since low blood carnitine levels have been shown to induce hyperammonemia, carnitine supplementation may potentially mitigate these symptoms.^[3]^ Although our patient did not manifest hyperammonemia, carnitine deficiency, or elevated VPA serum concentrations, neurological symptoms improved after VPA withdrawal. Therefore, when VPA-induced encephalopathy is suspected, VPA needs to be discontinued immediately, even if hyperammonemia or carnitine deficiency is not observed or the serum concentration of VPA is below the standard range.

## 4. Conclusion

We herein report a case of VPA-induced encephalopathy below the standard range of the serum concentration of VPA without hyperammonemia or carnitine deficiency. This condition may be caused by mechanisms other than hyperammonemia and l-carnitine deficiency. This diagnosis needs to be considered when patients treated with VPA exhibit neurological symptoms, even if their serum concentration of VPA is below the standard range, and they do not have hyperammonemia or carnitine deficiency.

## Acknowledgments

We would like to thank Medical English Service (https://www.med-english.com) for English language editing.

## Author contributions

**Conceptualization:** Jun Sawada, Tomohiro Uemura.

**Data curation:** Jun Sawada, Tomohiro Uemura, Nana Suzuki.

**Investigation:** Tomohiro Uemura, Nana Suzuki.

**Project administration:** Jun Sawada.

**Resources:** Jun Sawada, Tomohiro Uemura, Nana Suzuki, Jun Souma, Hikaru Kamiya, Shiori Takeguchi-Kikuchi, Tsukasa Saito.

**Supervision:** Jun Sawada, Naoki Nakagawa.

**Visualization:** Jun Sawada, Tomohiro Uemura.

**Writing – original draft:** Jun Sawada, Tomohiro Uemura.

**Writing – review & editing:** Naoki Nakagawa.
